# TOPICS ADDRESSED IN CENTENARIAN RESEARCH: A SCOPING REVIEW OF PUBLICATIONS SINCE 2000

**DOI:** 10.1093/geroni/igac059.2162

**Published:** 2022-12-20

**Authors:** Daniela Jopp, Charikleia Lampraki, Kim Uittenhove, Flavien Delhaes, Daniele Zaccaria, Carla Gomes Da Rocha, Garnelle Ziade, Melanie Becker

**Affiliations:** University of Lausanne, Lausanne, Vaud, Switzerland; University of Lausanne, Lausanne, Vaud, Switzerland; University of Lausanne, Lausanne, Vaud, Switzerland; University of Geneva, Geneva, Geneve, Switzerland; University of Applied Sciences and Arts of Southern Switzerland, Manno, Ticino, Switzerland; HES-SO Valais-Wallis, Sierre, Valais, Switzerland; University of Lausanne, Lausanne, Vaud, Switzerland; University of Zurich, Zurich, Zurich, Switzerland

## Abstract

Over the past decades, centenarians have increasingly attracted the attention of the research community. This development reflects the constant rise of the numbers of very old individuals, but also the need to better understand longevity mechanisms and what characterizes life at age 100. This scoping review provided an overview of trends in centenarian research from all disciplines since 2000, and identified the most frequently mentioned and neglected topics. Scientific articles meeting the following eligibility criteria were included: (1) publication between 2000 and 2021; (2) in English, French or German language; (3) study population of at least 95 years of age on average, or, for studies with larger age ranges, provision of specific insights for the 95+ group. Following the standard procedures for scoping reviews, we identified a total of N = 3955 articles. After removal of duplicates and exclusion due to unmet criteria, we used content coding to identify research themes of N = 1117 articles. Data confirmed that research articles offering findings on centenarians have increased substantially over the past two decades. Content coding led to 37 main topics: Most frequently, studies addressed higher-order topics such as physical, biological, and mental (e.g., cognitive), which were mostly investigated within disciplinary boundaries. Few multidisciplinary articles examined content domains in conjunction. The least investigated topics in centenarian research included the sub-domains of pain, stress, anxiety, and psychiatric disorders. In sum, findings inform the research community about the existing centenarian research, suggest that multidisciplinary publications are infrequent, and offer guidance for future studies.

